# Acute Phase Proteins and Vitamin D Seasonal Variation in End-Stage Renal Disease Patients

**DOI:** 10.3390/jcm9030807

**Published:** 2020-03-16

**Authors:** Małgorzata Maraj, Paulina Hetwer, Paulina Dumnicka, Piotr Ceranowicz, Małgorzata Mazur-Laskowska, Anna Ząbek-Adamska, Zygmunt Warzecha, Beata Kuśnierz-Cabala, Marek Kuźniewski

**Affiliations:** 1 Jagiellonian University Medical College, Faculty of Medicine, Department of Physiology, Grzegórzecka 16 St., 31-531 Kraków, Poland; malgorzata.maraj@doctoral.uj.edu.pl (M.M.); mpwarzec@cyf-kr.edu.pl (Z.W.); 2 Jagiellonian University Medical College, Faculty of Medicine, Dietetics, Anny St. 12, 31-008 Kraków, Poland; paulina.hetwer@op.pl; 3 Jagiellonian University Medical College, Faculty of Pharmacy, Department of Medical Diagnostics, Medyczna 9 St., 30-688 Kraków, Poland; 4 Diagnostic Department of University Hospital, Jakubowskiego 2 St., 30-688 Kraków, Poland; mbmazur@cyf-kr.edu.pl (M.M.-L.); azabek@su.krakow.pl (A.Z.-A.); 5 Jagiellonian University Medical College, Faculty of Medicine, Chair of Clinical Biochemistry, Department of Diagnostics, Kopernika 15A St., 31-501 Kraków, Poland; mbkusnie@cyf-kr.edu.pl; 6 Jagiellonian University Medical College, Faculty of Medicine, Chair and Department of Nephrology, Jakubowskiego 2 St., 30-688 Kraków, Poland; marek.kuzniewski@uj.edu.pl

**Keywords:** Vitamin D, hemodialysis, inflammation, acute phase proteins, diet, lifestyle

## Abstract

End-stage renal disease (ESRD) patients are vulnerable to vitamin D deficiency due to impaired renal hydroxylation, low dietary intake and inadequate sun exposure. Vitamin D plays a role in innate and adaptive immunity and its seasonal variation has been linked to mortality. ESRD is associated with inadequate removal of pro-inflammatory cytokines regulating acute phase protein (APP) synthesis. Our aim was to look for associations between lifestyle factors, diet, and vitamin D seasonal variation and their relationship with selected APPs and calcium-phosphate metabolism. The study included 59 ESRD patients treated with maintenance hemodialysis. A 24-hour dietary recall was conducted in the post-summer (November 2018, PS) and post-winter (February/March 2019, PW) period, and blood was collected for the measurements of serum total vitamin D, α_1_-acid glycoprotein (AGP), C-reactive protein (CRP), albumin, prealbumin (PRE), parathormone, calcium and phosphate. A self-constructed questionnaire gathered information on vitamin D supplementation, sun exposure and physical activity. Higher caloric intake was observed PW compared PS. Less than 15% of participants met the dietary recommendations for energy, protein, fiber, vitamin D and magnesium intake. Vitamin D supplementation was associated with higher serum vitamin D regardless of season. AGP, PRE, albumin, and vitamin D presented seasonal changes (higher values PS). In patients with serum vitamin D below 25 ng/mL, vitamin D seasonal change correlated with CRP and prealbumin change. Phosphate and Ca × P correlated positively with AGP. A low vitamin D serum level could impact the inflammatory process; however, more studies are needed to confirm the relationship.

## 1. Introduction

Vitamin D deficiency can affect up to 95% of end-stage renal disease (ESRD) patients treated with maintenance hemodialysis [1]. This, in part, is caused by a high concentration of fibroblast growth factor 23 (FGF-23), decreased activity of renal 1-α-hydroxylase, but also low dietary vitamin D intake and insufficient sun exposure [2]. It has been estimated that about 90% of vitamin D circulating in the serum derives from cutaneous skin synthesis in the presence of ultraviolet (UV) radiation [3]. In chronic kidney disease (CKD), uremia and hyperpigmentation, one of the most common cutaneous manifestations in hemodialysis (HD) patients, can additionally impair endogenous vitamin D synthesis [4]. According to Monitoring Dialysis Outcome (MONDO) data [5], mortality in hemodialysis patients is higher during the winter period in regions with significant difference in sun exposure between the summer and winter months. Numerous observational studies on ESRD patients have suggested that seasonality in vitamin D concentration can be linked to changes in bone turnover, but also vascular calcification and blood pressure [6,7,8,9,10,11,12]. Nonetheless, the interventional studies and meta-analyses of clinical studies failed to show the benefit of vitamin D supplementation in the reduction of cardiovascular events incidence or associated mortality, as well as all-cause mortality [13,14].

Vitamin D is primarily responsible for maintaining adequate levels of calcium in the serum, and its deficiency in CKD patients is associated with bone microarchitectural damage and decreased bone mineral density (BMD) [15]. The correction of vitamin D deficiency in ESRD patients is crucial in the management of kidney disease-related bone and mineral disorders and for the prevention of fractures and consequences of renal osteodystrophy [16,17,18]. 

ESRD is associated with chronic inflammation and often, malnutrition. Both these conditions have been reported to be associated with atherosclerosis and adverse cardiovascular outcomes [19,20]. In a multicenter observational study including Japanese ESRD patients treated with maintenance hemodialysis, the risk of all-cause mortality significantly increased with each additional factor of malnutrition-inflammation-atherosclerosis (MIA) syndrome [21]. Endothelial dysfunction and atherosclerosis have both been described in association with malnutrition and inflammation [19]. Malnutrition and inflammation remain a challenge in the clinical treatment of ESRD patients [22].

ESRD patients are exposed to multiple sources of inflammatory stimuli, such as volume overload, acidosis, co-morbidities, infections, and genetic and epigenetic influences, whereas a decreased glomerular filtration rate (GFR) leads to inadequate removal of pro-inflammatory cytokines [23]. Moreover, elevated FGF-23 in CKD acts as a promoter of inflammation and is associated with higher circulating levels of inflammatory cytokines [24], which can stimulate hepatic production of acute-phase proteins (APP). The mechanism for stimulation of hepatic production of acute-phase proteins (APP) is by proinflammatory cytokines—under the influence of interleukins (IL), such as IL-1, IL-2, IL-6, and the tumor necrosis factor-alpha (TNF-α), liver cells synthesize and secrete APP, which, among others, include albumin, prealbumin, ferritin, C-reactive protein (CRP), and α_1_-acid glycoprotein (AGP). The function of positive APP is regarded as important in the opsonization and trapping of microorganisms, activating the complement system, binding cellular remnants like nuclear fractions, neutralizing enzymes, scavenging free hemoglobin and radicals, and modulating the host’s immune response [25].

Vitamin D, on the other hand, down-regulates pro-inflammatory cytokines, including TNF-α, IL-1β, IL-6, and IL-8, and may be crucial for the anti-inflammatory response of monocytes [26]. It has been found that 1,25(OH)_2_D_3_ significantly reduced pro-inflammatory cytokines TNF-α, IFN-γ, and IL-1β, as well as the chemokine IL-8, while in stimulated monocytes, anti-inflammatory IL-10 increased twofold (*p* = 0.016) [27]. 

Vitamin D has an immunomodulatory function and can work indirectly via the regulation of transcription factors – evidence suggests that vitamin D suppresses the NFκB transcriptional activity, or regulates the cellular level of reactive oxygen species, which subsequently alter NFκB transcriptional activity [26]. Vitamin D receptors can be located on cell membranes (membrane vitamin D receptor, mVDR); however, most of vitamin D’s biological functions are mediated through the regulation of gene expression through binding to the nuclear receptor (nuclear vitamin D receptor, nVDR), which is found in multiple cells of the immune system, such as human T_reg_ cells, neutrophils, dendritic cells, B cells, and macrophages. Vitamin D can also directly influence the expression of genes with vitamin D response elements (VDRE) [28]. Particularly interesting seems the role of vitamin D in the stimulation of expression of α_1_-acid glycoprotein (AGP/Orsomucoid 1—ORM1) via the ORM1 gene characterized by the presence of functional VDRE [28]. AGP is an acute phase protein, a chronic inflammation marker which plays an anti-inflammatory function, suppressing neutrophil and complement activation [28,29]. It inhibits the proliferation of lymphocytes and its receptor has been found on macrophages. It is also responsible for maintaining the barrier function of capillaries and inhibition of platelet aggregation. It plays a protective role against infectious factors such as malaria, and could function as a non-specific competitor for cell surfaces, blocking the binding and invasion of infective agents [30,31]. AGP has been reported to have a protective effect against sepsis from gram-negative infections [30]. Moore et al. [32] showed that AGP interacts with the bacterial lipopolysaccharide (LPS), which is an initiator of the acute inflammatory response associated with septic shock, resulting in the formation of an AGP-LPS complex [33]. Hochepied et al. [30] demonstrated that AGP was effective against a lethal infection by *Klebsiella pneumonia,* and Shemyakinet al. [34] showed its preventive and therapeutic effect in mice against *Bacillus anthracis*.

The aim of the study was to find the relationships between the seasonal variations of vitamin D concentration and acute phase protein concentration changes, calcium-phosphate metabolism, and nutritional status markers in ESRD patients, reflecting the possible association of vitamin D seasonal changes with MIA syndrome frequently observed in such patients [35].

## 2. Materials and Methods

### 2.1. Participants

The prospective study included 59 adult patients in ESRD undergoing maintenance hemodialysis (three times a week), recruited from the Department of Nephrology of Jagiellonian University Medical College in Krakow, Poland. Participation in the study was voluntary, and written informed consent was obtained from all subjects. The study protocol had the approval of the Bioethics Committee (number 1072.6120.105.2018 issued on 20 April 2018). Data on comorbid diseases, the duration of treatment, and height and body mass were obtained from the hospital documentation. Data on drug treatment (phosphate binders, erythropoietin analogues, and iron) were verified based on hospital records.

The hemodialysis schedule included 4- to 5-hour sessions, three times a week. Patients were dialyzed using polysulphone dialyzers and bicarbonate dialysis fluid. Low-molecular heparin anticoagulation was used. The dialysis access was arteriovenous fistula in the majority of patients. The adequacy of dialysis was assessed by clinical evaluation and monthly Kt/V index evaluation (>1.2 in most patients). Only the patients with a stable dialysis course over at least two months were included in the study. 

### 2.2. Dietary Assessment and Questionnaire

A 24-hour dietary recall was conducted twice, first in November 2018, then in February/March 2019—each time, it inquired about the food intake during one working day and one of the weekend days. In order to obtain information on selected lifestyle aspects, including physical activity, diet, supplementation, daily time of sun exposure, and the surface area of the body exposed to solar radiation from April to October, a self-constructed questionnaire was distributed and completed by patients on their own or with the author’s assistance (P.H.).

### 2.3. Laboratory Tests

Laboratory tests were carried out in the Diagnostic Department of the University Hospital in Krakow, Poland. Patients’ blood samples were collected routinely for periodic examination—the blood was collected by puncturing the vein of the forearm to Sarstedt Company’s (Nümbrecht, Germany) closed systems according to evidence-based laboratory medicine. The measurements of selected parameters were carried out at the end of the summer season (post-summer (PS)—November 2018) and then repeated at the end of winter (post-winter (PW)—February/March 2019). For biochemical and immunochemical tests, serum was used. Serum total and ionized calcium, phosphates, total vitamin D (25OH-D_2_ and -D_3_), and intact parathyroid hormone concentrations were measured, as well as levels of acute phase proteins: albumin and C-reactive protein. Each assay was conducted with the use of an automated analyzer Cobas 8000 (Roche Diagnostic, Germany). In turn, measurements of prealbumin and α_1_-acid glycoprotein were carried out on an immunonephelometer BN II (Siemens, Germany). The degree of seasonal difference of the selected parameters was calculated by dividing the PS concentration value by PW concentration value and expressed as a percentage (PW/PS × 100%).

### 2.4. Nutritional Status Assessment

For the nutritional status assessment, standardized indicators were used: the cancer serum index (CSI), that is, the AGP/PRE ratio [36], the prognostic inflammatory and nutritional index (PINI) calculated according to the formula: CRP × AGP/PRE × ALB [37], as well as the CRP/PRE ratio [38].

### 2.5. Statistical Analysis

For the quantitative variables, the mean ± standard deviation and median (lower; upper quartiles) were shown depending on the normality of the distribution. The normality was assessed using the Shapiro–Wilk test. Differences between variables measured in the PS and PW periods were assessed with a *t*-test for paired variables or the Wilcoxon test, according to distribution. Differences between variables in two unrelated groups were assessed using the unpaired *t*-test or Mann–Whitney test. The Spearman rank coefficient was used to study correlations of vitamin D or acute phase protein concentrations and seasonal changes, as these variables were non-normally distributed. Multiple logistic regression was used to find independent predictors of vitamin D deficiency; the variables significantly associated with the deficiency in simple analysis were included in the model. Results were considered statistically significant at *p* < 0.05. The analysis was performed with Statistica 12.0 (StatSoft, Tulsa, OK, USA) software.

The sample size was based on the estimation that inclusion of 55 patients enables detection of 10% difference in repeated measurements of vitamin D and acute phase proteins (with an assumption of standard deviation equal to half of the mean) with a power of 90%, and that inclusion of 60 patients enables detection of a correlation of rho = 0.35 with a power of 80%, and a correlation of rho = 0.40 with a power of 90%.

## 3. Results

A total of 59 patients (36 men and 24 women) undergoing maintenance hemodialysis were recruited for the study. The average age of the participants was 58 years and the duration of renal replacement therapy ranged from 3 to 392 months. Nearly 80% of patients declared a low level of physical activity. Phosphate binders, mainly calcium carbonate, were used in almost 80% of the studied patients. Almost one half received erythropoiesis-stimulating agents, and about 40% received iron supplementation (Table 1).

Over 40% of patients used vitamin D supplementation (Table 1), where the recommended daily dose was 1000 IU (25 µg). Higher vitamin D concentration was characteristic for a subgroup supplementing vitamin D preparations regardless of the period: the median (lower; upper quartile) was 31.03 (20.02; 40.64) vs. 17.80 (13.58; 28.63) ng/mL in the PS period (*p* = 0.011) and 25.10 (16.40; 41.86) vs. 15.68 (10.80; 25.84) ng/mL in the PW assessment (*p* = 0.031). 

None of the studied patients received vitamin D analogues. Neither level of physical activity nor comorbidities (diabetes, hyperparathyroidism) differentiated the serum vitamin D level in the study group.

Among the studied patients, self-reported sun exposure in a period from April to October 2018 was moderate (Table 2). A numerical index (calculated as a sum of points received for answers to questions about sun exposure, as specified in Table 2) was constructed as a semi-quantitative measure of sun exposure, ranging from 3 (reflecting minimum exposure) to 9 points (high exposure). The median result in the studied group was 7. Only nine patients reported using creams or lotions with sun protection filters (SPF of 15 or higher). In the whole studied group, no significant associations were observed between sun exposure and vitamin D concentrations in PS period. However, a positive correlation was observed between the sun exposure index and PS vitamin D among subjects not receiving vitamin D supplementation (R = 0.41; *p* = 0.026). 

In the studied group, low levels of vitamin D (below 25 ng/mL) were observed in 33 (55.9%) patients in the post-summer period. In multiple logistic regression, only vitamin D supplementation negatively predicted vitamin D deficiency (odds ratio: 0.18; 95% confidence interval: 0.05–0.59; *p* = 0.004) independently of sun exposure.

The analysis of the 24-hour recall revealed that participants’ diets were highly nutritionally deficient. Less than 15% of participants met the daily nutritional recommendation for energy, proteins, fiber, vitamin D, and magnesium. No seasonal changes were observed in nutrient consumption except for a small but statistically significant increase in caloric intake in the PW compared with the PS period (Table 3). We observed no significant associations between nutrient consumption and serum concentrations of vitamin D. There were significant associations between protein intake and prealbumin (R = 0.28; *p* = 0.034), CRP (R = −0.29; *p* = 0.026), PINI (R = −0.30; *p* = 0.023), CSI (R = −0.27; *p* = 0.043), and CRP/PRE (R = −0.31; *p* = 0.021) in PS period and AGP (R = −0.40; *p* = 0.007) in the PW period. 

**Figure 1:**
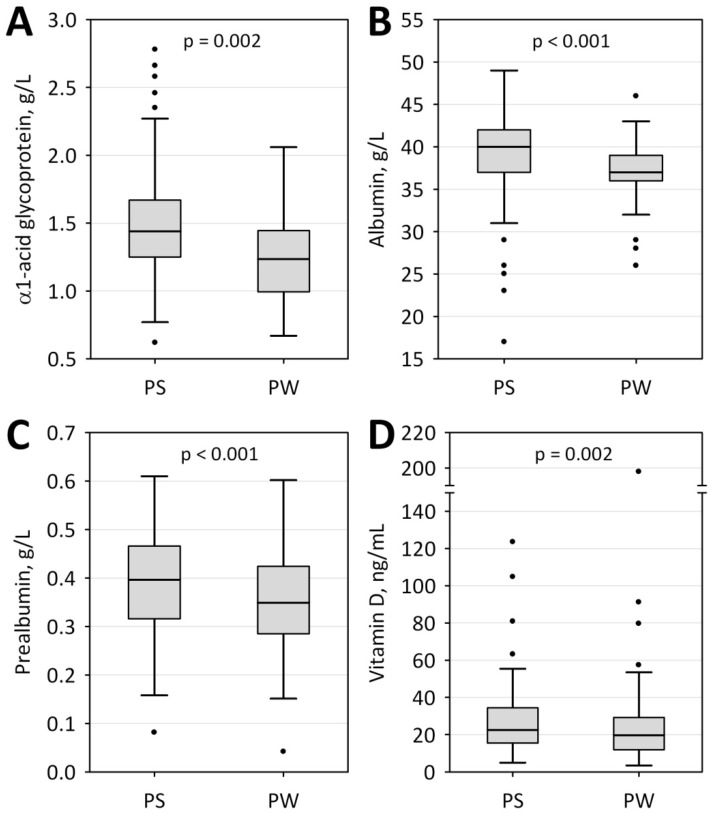
Changes in concentrations of selected parameters measured in post-summer (PS) and post–winter (PW) periods: α1- acid glycoprotein (**A**), albumin (**B**), prealbumin (**C**), and vitamin D (**D**). Data are shown as the median (central line), lower–upper quartile (box), non-outlier range (whiskers), and outliers (points).

In the studied group, we observed seasonal changes in concentrations of several acute phase proteins. The albumin, AGP, and prealbumin levels were significantly higher in the PS compared to PW period (Figure 1A–C). There were no statistically significant seasonal changes in CRP nor inflammatory and nutrition index values (PINI, CSI, CRP/PRE). Seasonal variability of serum vitamin D concentration was also demonstrated, although the parameters of calcium-phosphate status remained unchanged. Vitamin D concentrations were significantly higher in the PS compared to the PW period (Figure 1 D).

**Figure 2:**
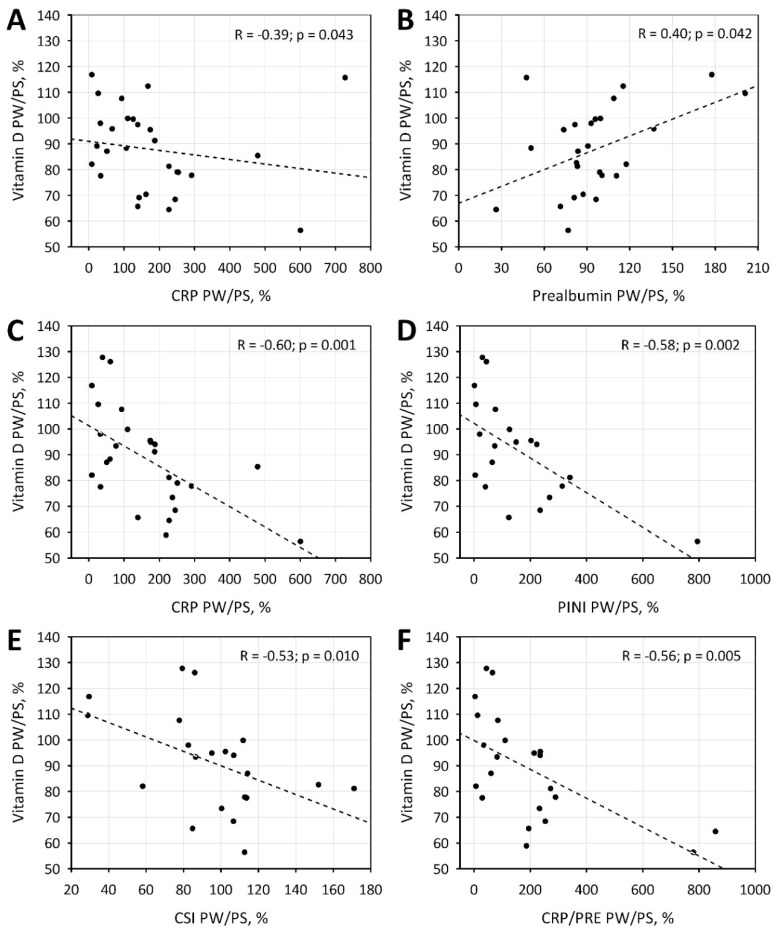
Correlations between seasonal changes in vitamin D and seasonal changes in C-reactive protein (CRP; **A**) and prealbumin (**B**) in patients with low initial concentrations of vitamin D (below 25 ng/mL). Correlations between seasonal changes in vitamin D and CRP (**C**), Prognostic Inflammatory and Nutritional Index (PINI; **D**), Cancer Serum Index (CSI; **E**) and CRP/prealbumin (CRP/PRE; **F**) in patients not receiving vitamin D supplementation.

In the whole studied group, the degree of seasonal change in vitamin D concentrations was not associated with the respective changes of the studied acute phase proteins, nutritional indexes, or mineral metabolism. We observed statistically significant correlations between the degree of seasonal changes in vitamin D and CRP and prealbumin concentrations (as well as CRP/PRE values: R = −0.45; *p* = 0.022) in participants with initial values of vitamin D lower than 25 ng/mL (Figure 2A,B). Moreover, the negative association between the seasonal changes in vitamin D and CRP, PINI, CSI, and CRP/PRE were also observed in a subgroup of patients not receiving vitamin D supplementation (Figure 2C–F). There was also a statistically significant negative correlation between the seasonal change of vitamin D and AGP concentrations in a subgroup of patients with an initial vitamin D concentration below 15 ng/mL.

**Figure 3:**
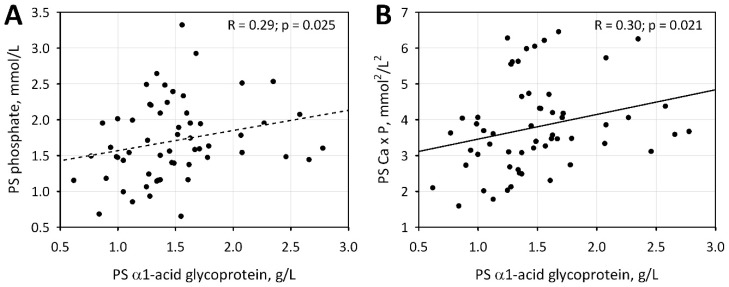
Correlations between α1- acid glycoprotein concentration and phosphate level (**A**) and Ca × P value (**B**) in the post-summer period.

Moreover, we demonstrated significant correlations between the AGP and phosphate concentration and Ca × P value, but only in samples obtained in the post-summer period (Figure 3). Both in the PS and PW periods, serum phosphate concentrations were positively correlated with phosphate intake (R = 0.30; *p* = 0.021 and R = 0.28; *p* = 0.044, respectively) and negatively associated with the use of phosphate binders (*p* = 0.010 and *p* = 0.025, respectively).

We did not observe significant associations between the use of erythropoiesis-stimulating agents or iron supplementation and the studied acute phase proteins or vitamin D. 

## 4. Discussion

### 4.1. Vitamin D Seasonal Variation

Patients in stage G5 of chronic kidney disease (CKD) are characterized by a low level of vitamin D in the serum due to impaired renal hydroxylation and low sun exposure. In the studied group, 69.5% of patients had a vitamin D concentration level below the lower reference range (<30 ng/mL). Regardless of season, patients taking supplementary vitamin D (42.4%) had a higher total serum vitamin D concentration than the non-supplementing ones (PS: *p* = 0.01, PW: *p* = 0.03). Moreover, seasonal changes of vitamin D concentration in the serum were observed—there was a statistically significant difference between the PS (November 2018) and PW (February/March 2019) periods (*p* = 0.002). This is in line with other studies [6,7,8,9,10,11,12] conducted in various geographical zones, including Central Europe, which reported a higher concentration of serum vitamin D in the spring and summer periods as compared to other seasons throughout the year. Vitamin D level is determined by sun exposure, but it may also be influenced by dietary vitamin D intake. In the present study, vitamin D supplementation was the most important factor determining serum 25(OH)D concentrations of ESRD patients, although the association between sun exposure and vitamin D serum concentrations was observed in a subgroup of patients who did not supplement vitamin D. The nutritional intake of vitamin D was much lower compared with the daily supplementation doses, and no significant association was observed between the nutritional intake and serum vitamin D.

In patients not supplementing vitamin D and those with low vitamin D concentrations, the seasonal variation of vitamin D was significantly associated with a seasonal variation of CRP and the indices reflecting the degree of inflammation and malnutrition. Moreover, in all studied patients, a positive correlation was also observed between the AGP concentration and phosphates and the Ca × P product. Vitamin D is directly connected with the calcium phosphate metabolism. The study by Yamada et al. [39] which was conducted on an animal model suggests that dietary phosphate overload induces systemic inflammation and malnutrition, accompanied by vascular calcification and premature death in CKD. 

### 4.2. Acute Phase Proteins’ Seasonal Variation

Low serum albumin concentration is common in ESRD patients and is associated with faster disease progression [40]. In the current study, we observed seasonal variation in the concentration of albumin and prealbumin—we recorded a significantly lower albumin concentration in the PW period as compared with the PS period; the same applied to prealbumin (Figure 1). Our results are the reverse of the results recorded by Yanai et al. [41] in the group of Japanese hemodialysis patients, who presented with a higher concentration of albumin in the winter period. Guinsburg et al. [5] in the study on international hemodialysis populations from the MONDO registry reported significantly higher albumin levels during winter in tropical climates in both hemispheres and in the northern temperate climate, but lower during winter in southern temperate climate. Since albumin is commonly used for the evaluation of nutritional status, it has been suggested that higher albumin concentration can be explained by higher consumption of nutrients in the winter [41]. We did not observe the correlation between protein intake and albumin concentrations, although the protein intake was positively associated with prealbumin levels in the PS period. In our study group, however, as much as 15.2% of patients had an albumin level below the lower reference range and its seasonal decrease may be connected with only a slightly higher caloric intake recorded in the PW period (Table 3), which still may have been insufficient to prevent the progression of malnutrition as the patients did not meet their daily dietary requirements regardless of season. The study by Gama-Axelsson et al. [42], however, does not confirm that albumin reflects the protein intake or the nutritional status in ESRD. It may as well be connected with chronic inflammation characteristic of ESRD, which occurs together with decreased albumin synthesis and intensified catabolic processes.

Prealbumin is another acute phase protein synthesized by the liver which decreases when protein and calorie intake is inadequate. In contrast to albumin, its half-life (about 2–3 days) is relatively short; therefore, it can reflect short-term dietary changes. Prealbumin, however, is not a reliable indicator of nutritional status for individuals with chronic renal failure—its levels may increase, presumably because of impaired degradation by the kidney. This could be the case in our study in which 45.8% of patients had prealbumin above the upper reference range. Prealbumin was higher at the end of the summer season, and it may not necessarily reflect the nutritional status of patients but may as well be connected with the seasonality of the inflammatory response. Similarly, to serum albumin, prealbumin has been shown to decrease in the face of acute and chronic inflammation. It must be noted, however, that although prealbumin may not correlate with changes in other nutritional parameters, it may be an independent mortality predictor [43,44]. 

To the best of our knowledge, for the first time we have shown the seasonality of AGP concentration in the serum of hemodialyzed patients—AGP concentration was higher in the PS period as compared to the PW period (*p* = 0.001). Vasson et al. [45] showed increased AGP concentration in the serum in patients with impaired renal function, including those which were hemodialyzed. In our study, 76% of patients had an initial AGP concentration above the upper reference limit. AGP concentration in the serum increases in response to stress- and inflammation-inducing factors [46]. It can play a protective function of tissue damage via the suppression of neutrophil activity and platelets aggregation [47]. The activity of AGP is, however, still not fully understood, and depending on its concentration and the cell it affects, it can have both a pro- and anti-inflammatory function [47]. The increased AGP expression facilitates LPS elimination, resulting in a protective effect against endotoxin shock derived from the infection [33]. At physiological concentrations, AGP, due to its antioxidant activity, protects erythrocytes from H_2_O_2_. According to these reports, an increase in the AGP content in serums above the normal value found under pathological conditions facilitates the passage of erythrocytes through capillaries, stabilizes erythrocyte membranes, and protects against oxidative stress, all of which are favorable properties for the microcirculation [33].

### 4.3. Relationship between Vitamin D and Acute Phase Proteins

Research referring to a correlation between vitamin D concentration and markers of inflammation are contradictory [48,49], but numerous studies point to the interdependence of vitamin D’s function and the inflammatory component. In our study, similarly to the vitamin D concentration changes, the albumin level was significantly lower in the PW period, but no correlation between the two was found. Nonetheless, Yonemura et al. [50] proved the relationship between albumin concentration and 25(OH)_2_D_3_ in ESRD patients. Vitamin D could potentially influence the albumin concentration via the activity decreasing the level of proinflammatory cytokines. It is suggested that the decrease in albumin concentration results from increased production of proinflammatory IL-6 and is a marker of inflammation [41]. Rao et al. [51] have shown the inverse correlation between IL-6 concentration and albumin in hemodialysis patients. Vitamin D has properties suppressing the expression of proinflammatory cytokines and interleukins, such as TNF-α, IL-1β, IL-2, IL-6, and IL-8 [26,52]. It can act via the receptors on the cell surface, but also via regulation of gene expression binding with VDRe (vitamin D response elements) on the surface of promotor regions [53], whereas nuclear vitamin D receptors are present on T lymphocytes, neutrophils, dendritic cells, and macrophages [26].

Our study revealed that vitamin D concentration changes between the PS and PW period inversely correlates with the CRP concentration change, but only in patients with an initial vitamin D level <25 ng/mL and those not supplementing vitamin D. This observation is in agreement with Meireles et al. [54] who showed that the vitamin D increase was a result of supplementation (from 14.3 ± 4.7 ng/mL to 43.1 ± 11.0 ng/mL) and significantly decreased CRP and IL-6 level in the serum of hemodialysis patients. Similarly, in the study by Ammar et al. [55], alpha-calcidiol supplementation during the period of 3 months increased the mean vitamin D concentration in the serum from 16.6 ng/mL to 38.95 ng/mL, simultaneously leading to the decrease in IL-6 and CRP concentration. This speaks for the anti-inflammatory effect of the vitamin D level serum increase in patients with its initial suboptimal level or deficit.

The statistical analysis also showed that in the group with an initial vitamin D concentration <15 mg/mL, its increase is associated with the decrease of AGP concentration. It must be noted, however, that the analyzed group of patients with vitamin D deficiency below 15 mg/mL is small (9 patients) and further studies are needed to confirm the relationship. AGP plays an immunomodulatory function in the course of chronic inflammation and tissue damage [47]. Gemelli et al. [28] have shown that there are anti-inflammatory properties of vitamin D of which AGP is an intermediary—produced not only in the liver, but also some immune cells, such as monocytes and macrophages. The AGP gene (ORM1) in the promotor region has VDRe, and the stimulation of 1,25(OH)D leads to the increase of its expression [28]. ORM1 activates the signaling pathway TLR4/CD14, decreasing the inflammation via the increase in the expression of inactivating molecules CD163 and MMP9 [28]. Perhaps the stimulation of AGP production by vitamin D depends on its initial concentration. It would be also important to identify factors modulating AGP concentration. 

Our study has several limitations. It was a single-center observational study and included a limited number of patients, allowing to detect moderate correlations (R = 0.35 to 0.40) with reasonable power. Weaker correlations between the studied variables might have been missed. The observed associations between seasonal variation in vitamin D and CRP, PRE, and the indices of malnutrition and inflammation (PINI, CSI, CRP/PRE) were significant only in a subgroup of patients, thus being too small to allow for multiple statistical analyses adjusted for confounders. It must be stressed that these associations cannot be interpreted as direct and may be an epiphenomenon caused by other factors. Moreover, the laboratory assessment the vitamin D status has not been standardized, and the results obtained by different tests may not be directly comparable.

## 5. Conclusions

In this study, levels of vitamin D, AGP, albumin, and prealbumin measured in the serum of patients treated with maintenance HD presented with seasonal changes. Vitamin D concentration changes between the post-winter and post-summer period was found to correlate with CRP and prealbumin change in HD patients with a vitamin D level <25 ng/mL and with CRP and inflammatory-nutritional indices in patients who did not use vitamin D supplementation. These results suggest a link between vitamin D status in ESRD patients on HD, and malnutrition and inflammation in patients with vitamin D deficiency; however, further studies are needed to verify the results and explore its clinical relevance.

## Figures and Tables

**Table 1:** Clinical characteristics and laboratory parameters of the study group recorded in the post-summer period. Quantitative data are shown as mean ± standard deviation or median (lower; upper quartiles).

Characteristics	The Study Group; *n* = 59
Age, years	57.9 ± 14.1
Female, *n* (%)	24 (40.7)
BMI, kg/m^2^	25.1 ± 5.3
Dialysis duration time, months	137 (39; 392)
Treatment with phosphate binders, *n* (%)	46 (77.9)
Calcium carbonate, *n* (%)	37 (62.7)
Aluminium hydroxide, *n* (%)	7 (11.9)
Sevelamer, *n* (%)	2 (3.4)
Vitamin D supplementation; *n* (%)	25 (42.4)
Treatment with erythropoietin analogues, *n* (%)	28 (47.5)
Epoietin, *n* (%)	14 (23.7)
Darbepoietin α, *n* (%)	6 (10.2)
Methoxy polyethylene glycol-epoetin β, *n* (%)	8 (13.6)
Low dose *, *n* (%)	21 (35.6)
High dose, *n* (%)	7 (11.9)
Iron supplementation, *n* (%)	25 (42.4)
Status post kidney transplantation; *n* (%)	12 (20.3)
Hyperparathyroidism; *n* (%)	13 (22)
Diabetes, *n* (%)	21 (35.6)
Physical activity level	
Low, *n* (%)	47 (79.7)
Moderate, *n* (%)	12 (20.3)
Vitamin D (ng/mL)	22.44 (15.59; 34.39)
Albumin (g/L)	40 (37; 42)
Prealbumin (g/L)	0.39 ± 0.11
CRP (mg/L)	3.16 (1.25; 9.27)
AGP (g/L)	1.44 (1.25; 1.67)
Total calcium (mmol/L)	2.19 (2.06; 2.41)
Phosphates (mmol/L)	1.70 ± 0.55
Intact PTH (pg/mL)	316.7 (119.5; 636.8)
Ca × P (mmol^2^/L^2^)	3.74 ± 1.26
PINI	0.29 (0.1; 1.23)
CSI	3.49 (2.70; 4.84)
CRP/PRE (×10^−3^)	7.57 (2.77; 12.79)
Hemoglobin (g/dL)	11.11 ± 1.15

* The following doses of erythropoietin analogues were categorized as low: <8000 IU/week of epoietin or <40 µg/week of darbepoietin α or ≤120 µg/month of methoxy polyethylene glycol-epoetin β. BMI: Body Mass Index, CRP: C-reactive protein, AGP: α_1_- acid glycoprotein, PRE: prealbumin, PTH: parathormone, Ca × P: calcium phosphate product, PINI: Prognostic Inflammatory and Nutrition Index, CSI: Cancer Serum Index.

**Table 2:** Sun exposure in a period from April to October, as reported by the studied patients.

Characteristics	Points	The Study Group; *n* = 59
Frequency of sun exposure:		
Every day, *n* (%)	3	37 (62.7)
3–4 times a week, *n* (%)	2	13 (22.0)
1–2 times a week or lower, *n* (%)	1	9 (15.2)
Duration of sun exposure session:		
≥30 min, *n* (%)	3	40 (67.8)
15–30 min, *n* (%)	2	9 (15.3)
<15 min, *n* (%)	1	10 (16.9)
Body surface exposed to sun:		
More than face, forearms and lower legs, *n* (%)	3	4 (6.8)
Face, forearms and lower legs, *n* (%)	2	40 (67.8)
Smaller, *n* (%)	1	15 (25.4)
Sun exposure index, points	sum of above	7 (6; 8)
Use of sun protection filter creams or lotions:		
Yes, *n* (%)	-	9 (15.2)
No, *n* (%)		50 (84.7)

**Table 3:** Dietary intake of selected nutrients in the post-summer and post-winter period. Data are shown as mean ± standard deviation or median (lower; upper quartiles).

	Post-Summer	Post-Winter	*p*
Energy (kcal/day)	1335 (1016; 1556)	1387 (1199; 1663)	0.043
Proteins (g/day)	51.0 ± 17.0	54.8 ± 16.4	0.1
Fat (g/day)	46.34 (36.41; 60.03)	48.09 (37.33; 60.99)	0.6
Carbohydrates (g/day)	184.2 (139.9; 225.9)	204.3 (152.7; 238.6)	0.09
Calcium (mg/day)	22.3 (170.8; 323.8)	249.1 (175.4; 355.7)	0.2
Phosphorus (mg/day)	711.2 (563.0; 853.6)	731.9 (655.4; 888.9)	0.5
Magnesium (mg/day)	169.61 ± 50.67	178.48 ± 65.61	0.4
Zinc (mg/day)	6.55 ± 2.27	6.88 ± 2.34	0.4
Potassium (mg/day)	1897.1 ± 626.9	1993.8 ± 675.7	0.9
Iron (mg/day)	7.07 ± 2.24	7.42 ± 2.31	0.3
Vitamin D (µg/day)	1.32 (0.80; 2.71)	1.44 (0.83; 2.58)	0.7
